# Assessing *in silico* the recruitment and functional spectrum of bacterial enzymes from secondary metabolism

**DOI:** 10.1186/s12862-017-0886-2

**Published:** 2017-01-26

**Authors:** Valery Veprinskiy, Leonhard Heizinger, Maximilian G. Plach, Rainer Merkl

**Affiliations:** 1 Institute of Biophysics and Physical Biochemistry, University of Regensburg, D-93040 Regensburg, Germany; 2 Faculty of Mathematics and Computer Science, University of Hagen, D-58084 Hagen, Germany

**Keywords:** Primary metabolism, Secondary metabolism, Enzyme evolution, Enzyme design

## Abstract

**Background:**

Microbes, plants, and fungi synthesize an enormous number of metabolites exhibiting rich chemical diversity. For a high-level classification, metabolism is subdivided into primary (PM) and secondary (SM) metabolism. SM products are often not essential for survival of the organism and it is generally assumed that SM enzymes stem from PM homologs.

**Results:**

We wanted to assess evolutionary relationships and function of *bona fide* bacterial PM and SM enzymes. Thus, we analyzed the content of 1010 biosynthetic gene clusters (BGCs) from the MIBiG dataset; the encoded bacterial enzymes served as representatives of SM. The content of 15 bacterial genomes known not to harbor BGCs served as a representation of PM. Enzymes were categorized on their EC number and for these enzyme functions, frequencies were determined. The comparison of PM/SM frequencies indicates a certain preference for hydrolases (EC class 3) and ligases (EC class 6) in PM and of oxidoreductases (EC class 1) and lyases (EC class 4) in SM.

Based on BLAST searches, we determined pairs of PM/SM homologs and their functional diversity. Oxidoreductases, transferases (EC class 2), lyases and isomerases (EC class 5) form a tightly interlinked network indicating that many protein folds can accommodate different functions in PM and SM. In contrast, the functional diversity of hydrolases and especially ligases is significantly limited in PM and SM.

For the most direct comparison of PM/SM homologs, we restricted for each BGC the search to the content of the genome it comes from. For each homologous hit, the contribution of the genomic neighborhood to metabolic pathways was summarized in BGC-specific html-pages that are interlinked with KEGG; this dataset can be downloaded from https://www.bioinf.ur.de.

**Conclusions:**

Only few reaction chemistries are overrepresented in bacterial SM and at least 55% of the enzymatic functions present in BGCs possess PM homologs. Many SM enzymes arose in PM and Nature utilized the evolvability of enzymes similarly to establish novel functions both in PM and SM. Future work aimed at the elucidation of evolutionary routes that have interconverted a PM enzyme into an SM homolog can profit from our BGC-specific annotations.

**Electronic supplementary material:**

The online version of this article (doi:10.1186/s12862-017-0886-2) contains supplementary material, which is available to authorized users.

## Background

Microbes synthesize a nearly astronomical number of metabolites that show rich chemical diversity and a broad range of biological activities. To achieve a high-level classification, Kossel introduced already in 1891 the term “secondary” to distinguish less relevant metabolites from “essential” ones, which he named “primary” [1]. In this way, he also coined the terms primary metabolism (PM) and secondary metabolism (SM). Since then, SM is defined as the sum of enzymatic reactions yielding natural compounds that are – in contrast to primary metabolites – not directly involved in growth, development or reproduction of an organism. Often, products of SM have an ecological function and serve as competitive weapons against other organisms, as agents of symbiosis or as sexual hormones [2]. Bacterial secondary metabolites are a rich source of antibiotics, chemotherapeutic drugs, and immune suppressants. Thus, they play important roles in medicine and are produced in large amounts by industrial microbiology. Nowadays, the complete genomic sequences of secondary metabolite producers are available and can be scanned rapidly to identify loci relevant for metabolite biosynthesis. Moreover, with recent genome editing and engineering techniques like CRISPR-Cas [3] allowing for rational pathway design, secondary metabolites are gaining new relevance [4].

Often, microbial secondary metabolites are derived by means of specific biosynthetic pathways and the corresponding genes are organized in biosynthetic gene clusters (BGCs) [5]. Compared to products of PM, secondary metabolites have a wider range of structures and biological activities [6]. This remarkable diversity reflects the random manner in which their biosynthesis has evolved. The pathways have been acquired opportunistically and horizontal gene transfer (HGT) of complete pathways concentrated in genomic islands is common [7]. However, horizontal gene transfer can only explain the propagation of already existing pathways but not the formation of the initial one; the latter process is unclear for most BGCs [8].

For the evolution of a novel SM pathway, it is generally assumed that it arises through the acquisition of genes from the PM repertoire [6, 9]. According to this theory, after initial gene duplication of the PM predecessor, subsequent mutations shape the biological activity of the gene copy in a way that may give rise to “abnormal” products. If not directly beneficial, these products might become so after spontaneous chemical change or after modifications by other enzymes with broad substrate spectra, which may eventually result in a strain with a selective advantage [6]. Nowadays it is feasible to identify for an SM enzyme the primary precursor by means of computational biology and to confirm the most likely evolutionary route with the help of biochemical experiments. Thus, having chosen for a given SM enzyme the most likely PM predecessor, one can estimate the evolutionary cost needed for the genesis of a novel enzymatic function utilized in SM.

For example, chorismate is a central metabolic branch point molecule and the common precursor of primary (folate, tryptophan) and secondary metabolites (menaquinones, siderophores, antibiotics), which are vital for plants as well as free living and infectious microorganisms [10]. In PM, aminodeoxychorismate synthase (ADCS, folate biosynthesis) and anthranilate synthase (AS, tryptophan biosynthesis) form aminated chorismate derivatives. Both are heteromeric complexes consisting of the enzymes PabA/PabB or TrpG/TrpE, respectively. In SM, isochorismate synthase (ICS) hydroxylates chorismate for the synthesis of menaquinones and siderophores and is a homolog of PabB and TrpE [11]. We have recently reported on the biochemical conversion of an AS into an ICS by altering the nucleophile specificity of AS from ammonia to water. Interestingly, not more than two amino acid exchanges in a channel leading to the catalytic site were sufficient to interconvert AS into a bifunctional AS/ICS that can be utilized in SM [11].

The generally accepted hypothesis for BGC genesis, which assumes the recruitment of PM enzymes, is so far only based on the analysis of few enzyme families. For example, polyketide synthases (PKSs) and nonribosomal peptide synthases (NRPSs) have been traced back to their PM homologs [7, 8, 12]. However, a comprehensive compilation of such pairs of homologs it still missing. To fill this gap, we browsed the content of SM databases and identified homologous enzymes known to contribute to PM. We focused on metabolic enzymes from bacteria because their genomes are extensively annotated, which is a prerequisite for a detailed analysis. The enzyme pairs that we identified can be used now to elucidate modifications introduced by evolution in a PM enzyme to serve in SM and to guide conversion experiments similar to the one described above. Moreover, we characterized the evolvability of enzymes and the range of enzymatic functions occurring in bacterial SM and made plausible that for a minimum of 331 enzyme functions homologs occur both in PM and SM.

## Results

### A compilation of *bona fide* PM and SM enzymes

Unfortunately, the discrimination of PM and SM introduced by Kossel did not rely on function and was a purely phenomenological definition [8]. For example, lipids or polysaccharides are “essential” for every organism, but the synthesis of some of them is specific for a small class of species [9]. Therefore, it is often difficult to assign a metabolic pathway or an enzyme function exclusively to PM or SM. Databases like BRENDA [13] do not assign enzymes to PM or SM and the KEGG database [14] classifies too many enzymes as SM: For example, tryptophan biosynthesis belongs to PM in bacteria; however, the related gene products are annotated by KEGG as SM. It follows that one has to restrict the analysis to a carefully chosen subset, if one is interested to study enzymes, whose assignment to PM is without any doubt.

C. Hertweck and co-workers analyzed 211 complete genomes of anaerobic bacteria and identified 26 species that do not contain BGCs [15]. Among these 26 genomes lacking SM, we selected those that are integrated into KEGG, because we were dependent on a comprehensive annotation and we thus opted for the KEGG and BRENDA databases. In order to reduce phylogenetic bias, we eliminated closely related species; the names of the 15 remaining ones are listed in Table 1. The annotations of these genomes were scanned to identify enzymes; the related 20370 sequences were added to the set *enzymes*_PM*_. Note that we use the label “PM*”, in order to explicitly indicate that we analyzed a specific and possibly uncomplete subset of enzyme functions contributing to PM.

**Table 1:** Bacterial species known not to contain secondary metabolite gene clusters

Bacterial species and description	Tax-ID
*Dehalococcoides* sp. VS Chloroflexi (ph), Dehalococcoidia (cl), Dehalococcoidales (or), Dehalococcoidaceae (fa), Dehalococcoides (gn) Anaerobic, obligately organohalide-respiring	311424
*Dehalogenimonas lykanthroporepellens* BL-DC-9 Chloroflexi (ph), Dehalococcoidia (cl), Dehalogenimonas (gn) Strictly anaerobic, mesophilic, non spore-forming, Gram-negative	552811
*Chloroflexus aurantiacus* J-10-fl Chloroflexi (ph), Chloroflexia (cl), Chloroflexales (or), Chloroflexaceae (fa), Chloroflexus (gn) Filamentous anoxygenic phototroph, thermophilic green bacterium	324602
*Deferribacter desulfuricans* SSM1 Deferribacteres (ph), Deferribacterales (or), Deferribacteraceae (fa), Deferribacter (gn) Strictly anaerobic, thermophilic, sulphur-reducing, heterotroph	639282
*Calditerrivibrio nitroreducens* Yu37-1 Deferribacteres (ph), Deferribacterales (or), Deferribacteraceae (fa) Strictly anaerobic, moderately thermophilic, nitrate-reducing, Gram-negative, non-sporulating	768670
*Denitrovibrio acetiphilus* N2460 Deferribacteres (ph), Deferribacterales (or), Deferribacteraceae (fa), Denitrovibrio (gn) Obligately anaerobic, mesophilic, nitrate reducing	522772
*Flexistipes sinusarabici* MAS10 Deferribacteres (ph), Deferribacterales (or), Deferribacteraceae (fa), Flexistipes (gn) Strictly anaerobic, moderately thermophilic, Gram-negative, non-motile, heterotrophic, marine habitat	717231
*Desulfurispirillum indicum* S5 Chrysiogenetes (ph), Chrysiogenales (or), Chrysiogenaceae (fa), Desulfurispirillum (gn) Strictly anaerobic, uses selenate, selenite, arsenate, nitrate or nitrite as terminal electron acceptors	653733
*Thermodesulfatator indicus* CIR 29812 Thermodesulfobacteria (ph), Thermodesulfobacteriales (or), Thermodesulfobacteriaceae (fa), Thermodesulfatator (gn) Anaerobic, thermophilic, chemolithoautotrophic sulfate reducer isolated from a deep-sea hydrothermal vent	667014
*Thermanaerovibrio acidaminovorans* Su883 Synergistetes (ph), Synergistia (cl), Synergistales (or), Synergistaceae (fa), Thermanaerovibrio (gn) Anaerobic, isolated from an reactor of a sugar refinery, Gram-negative, motile, non-spore-forming	525903
*Aminobacterium colombiense* ALA-1 Synergistetes (ph), Synergistia (cl), Synergistales (or), Synergistaceae (fa), Aminobacterium (gn) Isolated from an anaerobic lagoon, mesophilic, amino acid fermenting, Gram-negative, non-sporulating	572547
*Thermovirga lienii* Cas60314 Synergistetes (ph), Synergistia (cl), Synergistales (or), Synergistaceae (fa), Thermovirga (gn) Anaerobic, thermophilic, chemoorganotrophic, Gram-negative, motile, from a marine oil well	580340
*Akkermansia muciniphila* ATCC BAA-835 Verrucomicrobia (ph), Verrucomicrobiae (cl), Verrucomicrobiales (or), Akkermansiaceae (fa), Akkermansia (gn) Anaerobic, isolated from the human intestinal tract	349741
*Thermus scotoductus* SA-01 Deinococcus-Thermus (ph), Deinococci (cl), Thermales (or), Thermaceae (fa), Thermus (gn) Growth with oxygen and nitrate as terminal electron acceptors, reduces a variety of metal ions	743525
Candidatus *Cloacamonas acidaminovorans* Candidatus Cloacimonetes (ph), Candidatus Cloacimonas (gn) Anaerobic digester of a municipal wastewater treatment plant	459349

The respective genomes are part of KEGG databases. The name, the NCBI Tax-ID and the phylogenetic lineage are listed; abbreviations are: phylum (ph), class (cl), order (or), family (fa), genus (gn). Additionally, a short description of the habitat and of the species are given, which were taken from [19]

Following the above arguments, we also had to restrict the analysis of SM enzymes by choosing well characterized cases. The most comprehensive compilation of microbial SM is the dataset *Minimum Information about a Biosynthetic Gene cluster* (MIBiG) [5]; each cluster has been individually annotated by experts in their fields. We analyzed 1010 bacterial BGCs of MIBiG (version 1) that contained 18390 proteins and identified 2724 enzymes with a precisely specified function. We named this set *enzymes*_SM*_ to indicate that we selected a well-defined, but restricted subset of SM pathways.

### The enzymatic spectra of bacterial SM and PM overlap to a great extent

The entries and annotations of the sets *enzymes*_PM*_ and *enzymes*_SM*_ were used to estimate enzymatic capabilities of PM and SM. To begin with, we determined for all entries the assigned EC numbers (*EC_*#) [16], because they specify unequivocally the catalyzed reactions. Moreover, EC numbers are organized in a hierarchical manner, which can be used to group similar reactions.

The first digit of each EC number is a class number (*EC_cl*) indicating one of six types of chemical reactions. The class EC 1 subsumes oxidoreductases that catalyze oxidation/reduction reactions and EC 2 transferases that transfer functional groups. EC 3 consists of hydrolases that catalyze the formation of two products from a substrate by hydrolysis and EC 4 contains lyases that catalyze the non-hydrolytic addition or removal of groups. The isomerases of EC 5 catalyze the intramolecular rearrangement within a single molecule and the ligases of EC 6 join together two molecules under consumption of ATP or similar triphosphates [17].

The second and third digits subdivide the reactions into subclasses (*EC_sc*) and subdivisions (*EC_sd*). The fourth digit is a serial number and addresses the substrate. Thus, if the first three digits of EC numbers are identical, the considered gene products belong to the same subdivision, *i. e*. share the same reaction chemistry. As we were interested to assess the occurrence of more general functions, we grouped enzymes on the first or up to the third EC digits, which is a common approach [18].

The 20370 entries of *enzymes*_PM*_ have assigned 1197 different EC numbers. The normalized frequencies *f*_*PM* *_(*EC*_ #) were combined to assess the occurrence of more general reaction chemistries. Analogously, we determined normalized frequencies *f*_*SM* *_(*EC*_ #) and combined corresponding values. Table 2 lists these frequencies for EC classes, and Additional file 1: Table S1 those of subclasses, subdivisions, and of all EC numbers.

**Table 2:** Abundance of EC classes in *enzymes*_PM*_ and *enzymes*_SM*_

EC Class	Enzyme Function	*f*_*PM* *_	*f*_*SM* *_	*f*_*Ecoli*_	*f*_*Myco*_
1	Oxidoreductases	18.71	22.10	19.86	8.38
2	Transferases	35.25	36.33	33.57	41.32
3	Hydrolases	17.25	11.64	25.13	22.16
4	Lyases	10.70	17.28	9.45	2.99
5	Isomerases	6.67	6.81	6.41	8.38
6	Ligases	11.42	5.84	5.58	16.77

*f*_*PM* *_- and *f*_*SM* *_-values are the normalized frequencies for the occurrence of EC classes in the datasets *enzymes*_PM*_ and *enzymes*_SM*_ *. f*_*Ecoli*_ and *f*_*Myco*_ are the frequencies of EC classes deduced from the genomes of *E. coli* and *M. genitalium*

The SM* enzymes have assigned 600 EC numbers. 331 of these enzyme functions occur both in PM* and SM* and 269 were exclusively found in *enzymes*_SM*_. On the other hand, *enzymes*_PM*_ catalyze 866 specific functions not found in *enzymes*_SM*_. The enzyme functions of SM* belong to 123 different subdivisions. Not more than 13 of these subdivisions do not occur in *enzymes*_PM*_ and ten of them are oxidoreductases. All SM* frequencies can be found in Additional file 2: Table S2.

### Oxidoreductases and few other enzymes are key components of bacterial BGCs

The comparison of the EC class frequencies listed in Table 2 shows that the classes EC 2 (transferases) and EC 5 (isomerases) are approximately equally abundant in *enzymes*_PM*_ and *enzymes*_SM*_. The classes EC 3 (hydrolases) and EC 6 (ligases) are underrepresented to a certain degree and EC 1 (oxidoreductases) and EC 4 (lyases) are overrepresented in *enzymes*_SM*_.

Why are oxidoreductases that catalyze oxidation/reduction reactions, overrepresented in SM? Most of the species used to compile the set *enzymes*_PM*_ live in anaerobic habitats. As a consequence, the low frequency *f*_*PM* *_(*EC*_*cl*) of oxidoreductases could be an artefact caused by a biased selection of PM enzymes in *enzymes*_PM*_. In order to rule out a sampling bias and to further assess the effect of genome size on *f*_*PM* *_(*EC*_*cl*), we analyzed the *Escherichia coli* MG1655 genome (KEGG T00007) and that of *Mycoplasma genitalium* G37 (KEGG T00002). Both species are able to grow aerobically and anaerobically and *M. genitalium* is thought to have the smallest genome of any self-replicating organism [19]. Although all abundancies vary noticeably, the frequency of encoded oxidoreductases is for both species smaller than in *enzymes*_SM*_, which argues for a certain overrepresentation of oxidoreductases in bacterial SM and against a sampling bias in *enzymes*_PM*_. This conclusion is in agreement with the known high SM abundance of oxygenases [20] and reflects that oxygen is a prerequisite for the synthesis of alkaloids and special antibiotics [21]. In a similar manner, lyases are more abundant in *enzymes*_SM*_ than in *enzymes*_PM*_ and in the genomes of *E. coli* and *M. genitalium*; however, this bias is unclear to us.

**Fig. 1:**
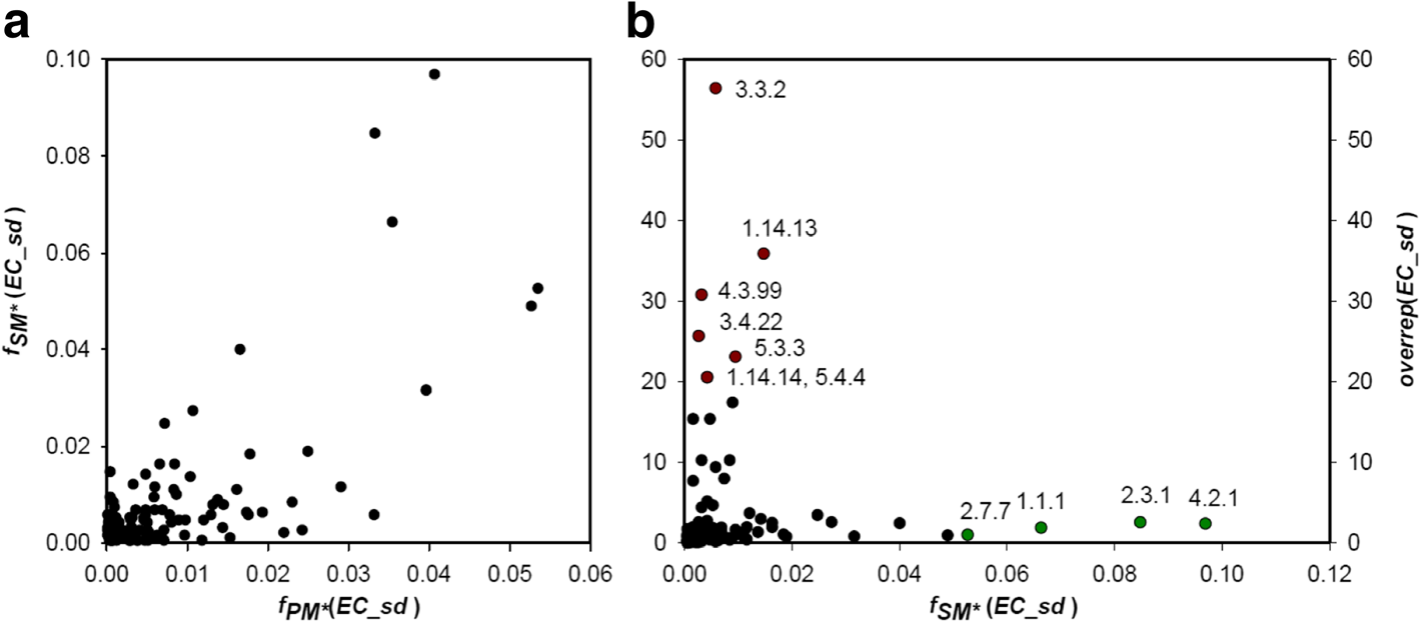
Occurrence of EC subdivisions in PM* and SM* and their overrepresentation in BGCs. **a** A plot of *f*_*PM* *_(*EC*_*sd*) -values versus *f*_*SM* *_(*EC*_*sd*) -values. These are the normalized frequencies for the occurrence of EC subdivisions in the datasets *enzymes*_PM*_ and *enzymes*_SM*_, respectively*.* **b** A plot of *overrep*(*EC_sd*)-values versus *f*_*SM* *_(*EC*_*sd*) -values. Each *overrep*(*EC_sd*)-value is the ratio *f*_*SM* *_(*EC*_*sd*)/*f*_*PM* *_(*EC*_*sd*) that relates the abundance of a subdivision in *enzymes*_PM*_ and *enzymes*_SM*_. For subdivisions with an *overrep*()-value > 20 (red symbols) and those most abundant in *enzymes*_SM*_ (green symbols), the *EC_sd* number is given

For a more detailed analysis of functional spectra, we compared the frequencies of EC subdivisions (*i. e*. reaction chemistries). Panel a of Fig. 1 is a plot of *f*_*PM* *_(*EC*_*sd*) - versus *f*_*SM* *_(*EC*_*sd*) -values. Interestingly, the corresponding frequency pairs are moderately correlated with *r* ^2^ = 0.51, indicating that many functions occur in PM* and SM* with similar frequencies. Additionally, we determined the ratio *overrep*(*EC*_*sd*) = *f*_*SM* *_(*EC*_*sd*)/*f*_*PM* *_(*EC*_*sd*). Panel b of Fig. 1 shows that those subdivisions that are strongest overrepresented are also rare in *enzymes*_SM*_ and the *overrep*-values indicate an even lower abundance in *enzymes*_PM*_. The most prominent subdivisions belong to EC 3.3.2 (ether hydrolases), EC 1.14.13 and EC 1.14.14 (oxidoreductases, acting on paired donors), EC 3.4.22 (cysteine endopeptidases), EC 4.3.99 (other carbon-nitrogen lyases), EC 5.3.3 (intramolecular oxidoreductases), and EC 5.4.4 (isomerases, transferring hydroxy groups). The corresponding enzyme functions are related to known key elements of SM, namely oxygen transfer, ether synthesis, or the nonribosomal biosynthesis of peptides [22]. On the other hand, the subdivisions that are most abundant in *enzymes*_SM*_ with *f*_*SM* *_(*EC*_*sd*) > 0.05, namely EC 4.2.1 (hydrolyases), EC 2.3.1 (acyltransferases, transferring groups other than amino-acyl groups), EC 1.1.1 (oxidoreductases, acting on the CH-OH group of donors with NAD+ or NADP+ as acceptor), and EC 2.7.7 (nucleotidyltransferases) occur in *enzymes*_PM*_ with similar frequencies (*overrep* () ≈ 1). In summary, our findings suggest that the range of reaction chemistries used in PM* and SM* overlap to a great extent and that only few enzymes are highly specific for SM*.

### The distribution of monofunctional and multifunctional families is similar in *enzymes*_PM*_ and *enzymes*_SM*_

The above approach made plausible that approximately 55% of the enzymatic functions observed in *enzymes*_SM*_ are also present in *enzymes*_PM*_. However, due to convergent evolution, enzymes that catalyze the same reaction do not necessarily possess the same 3D structure. We wanted to know whether these joint PM/SM enzyme functions have been established on the same or different protein folds.

Sequence alignments unambiguously distinguish proteins possessing similar and non-similar structures [23] and as stated by W.R. Pearson, homology of two protein sequences can be reliably inferred from a statistical significant BLAST hit [24]. In the following, we use homology as coined by W.R. Pearson as a term for similar structure (*i. e*. identical fold) and common ancestry. Tracing the line of descent in more detail is difficult because BGCs and other SM functions are frequently acquired via horizontal gene transfer [7]. Thus, it is hard to decide whether the gene copies arose via speciation (orthologs) or gene duplication (paralogs) and whether the acquisition or gene genesis is a more recent or ancestral event.

We used blastp with the stringent cutoff 1E-20 and searched in *enzymes*_PM*_ for hits related to enzymes from *enzymes*_SM*_. These PM*/SM* enzymes were considered as homologs. In order to eliminate false positive hits due to only one or few shared domains in multi-domain enzymes, we considered for each enzyme from *enzymes*_SM*_ only those *enzymes*_PM*_ hits that differed in length not more than 30% from the query.

For 269 enzyme functions from 27 subdivisions, we did not find a homolog in *enzymes*_PM*_. 81 of the *enzymes*_SM*_-only functions are oxidoreductases (EC class 1); this finding supports their SM* overrepresentation determined above. However, our main goal was to characterize cases of SM* enzymes that can be traced back to PM* enzymes. Therefore, we concentrated on those SM* enzyme functions, for which BLAST found at least one PM* hit. These were 331 enzyme functions from 96 subdivisions; thus we could significantly increase the set of SM enzymatic functions for which an origin in PM can be taken for granted.

These PM*/SM* homologs are not necessarily isofunctional, because even a BLAST E-value below 1E-50 does not guarantee that the two compared sequences encode the same protein function [25]. For example, ICS (EC 5.4.4.2), PabB (EC 2.6.1.85), and TrpE (EC 4.1.3.27) are homologous [11] although their functions belong to three different EC classes. We were interested in assessing the rate of SM* enzymes whose PM* homologs catalyze different functions. This is why we compared for all EC subdivisions the EC numbers of *enzymes*_SM*_ and their homologs in *enzymes*_PM*_. For 48 cases, the SM* enzymes and all of their PM* homologs share the same subdivision. For 45 cases, PM* homologs are from at least two different subdivisions, and for 3 SM* subdivisions, all PM* homologs belong to a completely different subdivision. These findings indicate that approximately 39% (48/123) of these SM* subdivisions are constituted by duplicated enzymes that utilize only one reaction chemistry both in *enzymes*_PM*_ and *enzymes*_SM*_. On the other hand, for 37% (45/123) of these SM* subdivisions, their members belong to enzyme families that support in PM* a larger spectrum of functions. The determined fraction of multifunctionality is a conservative approximation: When applying the cutoff 1E-10, the number of monofunctional SM*/PM* enzyme pairs (same EC subdivision) decreased to 42, and that of multifunctional ones increased to 52. However, lowering the stringency of this cutoff increases the risk of predicting false positives. As we were interested to identify highly reliable relationships, we utilized for the following analyses the conservatively chosen cutoff 1E-20.

To estimate in more detail the number of identical PM*/SM* functions, we compared the full EC numbers. Of the 600 EC numbers under study, the SM* queries and their PM* hits had the same number for 154 cases. Homologs with different EC numbers were found for 119 cases, and for 58 cases the EC number of all PM* hits differed from the EC number of the SM* query. Thus, of the 331 enzyme functions that occur both in *enzymes*_PM*_ and *enzymes*_SM*_, 46% belong to monofunctional families, 36% to multi-functional families and 18% most likely changed their function after recruitment from PM.

Are these three fractions to be expected? For a comparison, we BLASTed with the same parameters all enzymes $enzyme{s}_{genom{ e}_i}$ from each of the 15 genomes *genome*_*i*_ constituting *enzymes*_PM*_ against the specific set $\left\{ e nzyme{s}_{PM*}\backslash enzyme{s}_{genom{ e}_i}\right\}$ that lacks the content of $enzyme{s}_{genom{ e}_i}$, *i. e.* the PM* enzymes found in one genome. 41% of the PM*/PM* BLAST hits belonged to monofunctional enzyme families, 48% to multi-functional families and for 11% all hits had a different EC number. Analogously, the comparison of each set $enzyme{s}_{BG{ C}_i}$ (enzymes from one BGC) against $\left\{ enzyme{s}_{SM*}\backslash enzyme{s}_{BG{ C}_i}\right\}$ (content of all other BGCs) gave 41% monofunctional, 34% multi-functional enzyme families and 25% of the hits had a different EC number. The comparisons of the corresponding fraction values (PM*/PM* versus PM*/SM* or SM*/SM*) indicate that the degree of neofunctionalization is similar in PM* and SM*.

### Recruited SM enzymes reveal a typical pattern of functional flexibility

It is known that a large portion (71%) of all enzyme functions is performed by a relatively small set of 276 superfamilies [26]. Comparing the function of the corresponding members, it was shown that during enzyme evolution, 85% of functional changes led to enzymes belonging to the same EC class. The remaining 15% of the novel enzyme functions led to a change between EC classes. In 70% of these cases, enzymes from the EC classes 1, 2, and 3 were involved, and changes between isomerases and lyases (EC 4↔EC 5) were more frequent than expected [27].

We were interested to determine the functional flexibility of those enzymes that were recruited for SM and thus we related the EC numbers EC_#_SM* occurring in *enzymes*_SM*_ and the EC numbers EC_#_PM* of their homologous BLAST hits from *enzymes*_PM*_. These abundancies were summarized on the level of EC classes and subdivisions, respectively, and for the corresponding BLAST E-values the mean was determined. Using Cytoscape [28], a network was created in which EC classes or subdivisions were represented by nodes and the number of PM hits and their mean E-values were used to determine width and color of interconnecting edges. These edges indicate a functional change (PM → SM), because PM homologs possess a different function. Additionally, we determined the rate of functional conservation *fc*(*EC_cl*) by computing the fraction of PM* homologs that belong to the same EC class or subdivision as the SM* queries.

**Fig. 2:**
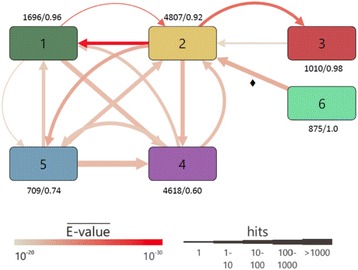
Multifunctionality deduced from homologous PM*/SM* pairs and determined for EC classes. The nodes represent the six EC classes and arrows indicate the relation of functional difference PM* → SM*. For example, the arrow 6 → 2 signals that PM* homologs of SM* class 2 enzymes belong to EC class 6; this arrow is marked with a ♦ symbol. The width of the arrows represents the number of BLAST hits of enzymes from *enzymes*_SM*_ in *enzymes*_PM*_ and their color the mean E-value; hits were binned as indicated. In addition, for each class, the number of PM* BLAST hits is given and the rate of functional conservation *fc*, which is the fraction of PM* BLAST hits that belong to the same EC class as the SM* queries. The class EC 1 subsumes oxidoreductases that catalyze oxidation/reduction reactions and EC 2 transferases that transfer functional groups. EC 3 consists of hydrolases that catalyze the formation of two products from a substrate by hydrolysis and EC 4 contains lyases that catalyze the non-hydrolytic addition or removal of groups. The isomerases of EC 5 catalyze the intramolecular rearrangement within a single molecule and the ligases of EC 6 join together two molecules under consumption of ATP or similar triphosphates

Figure 2 highlights three major trends on the class level: *i*) Oxidoreductases (EC class 1), transferases (EC class 2), lyases (EC class 4), and isomerases (EC class 5) form a tightly interlinked network indicating that many of these enzymes (*i. e.* folds) can adopt different functions in PM and SM. Among them, PM homologs of SM transferases support the widest functional spectrum indicated by the five edges ending in EC class 2. The functional conservation *fc*(*EC_cl*) was below 0.9 only for lyases (*fc* = 0.74) and for isomerases (*fc* = 0.60) indicating that a large fraction of the homologs catalyze completely different reactions in PM*. *ii*) In contrast, for SM* hydrolases (EC class 3) the functional flexibility of PM* homologs is limited to transferases (EC class 2). Thus, although hydrolases are abundant in *enzymes*_PM*_ and *enzymes*_SM*_ (Table 2), few are from multifunctional families, which may be due to their special chemistry of cleaving bonds by adding H_2_O. *iii*) None of the PM* homologs of SM* ligases (EC class 6) had a function belonging to a different EC class. Ligases catalyze the joining of two molecules by hydrolyzing ATP or other triphosphates. It seems difficult to integrate this functionality into scaffolds from EC class 1 - 5 enzymes. The limited flexibility of EC class 3 and EC class 6 enzymes is not an artefact caused by a too stringent cutoff. We lowered the BLAST cutoff to 1E-10 and repeated the analysis of functional flexibility. The resulting graph is shown in Additional file 3: Figure S1. It contains only one additional arrow (6 → 2), which is compatible with the above findings.

**Fig. 3:**
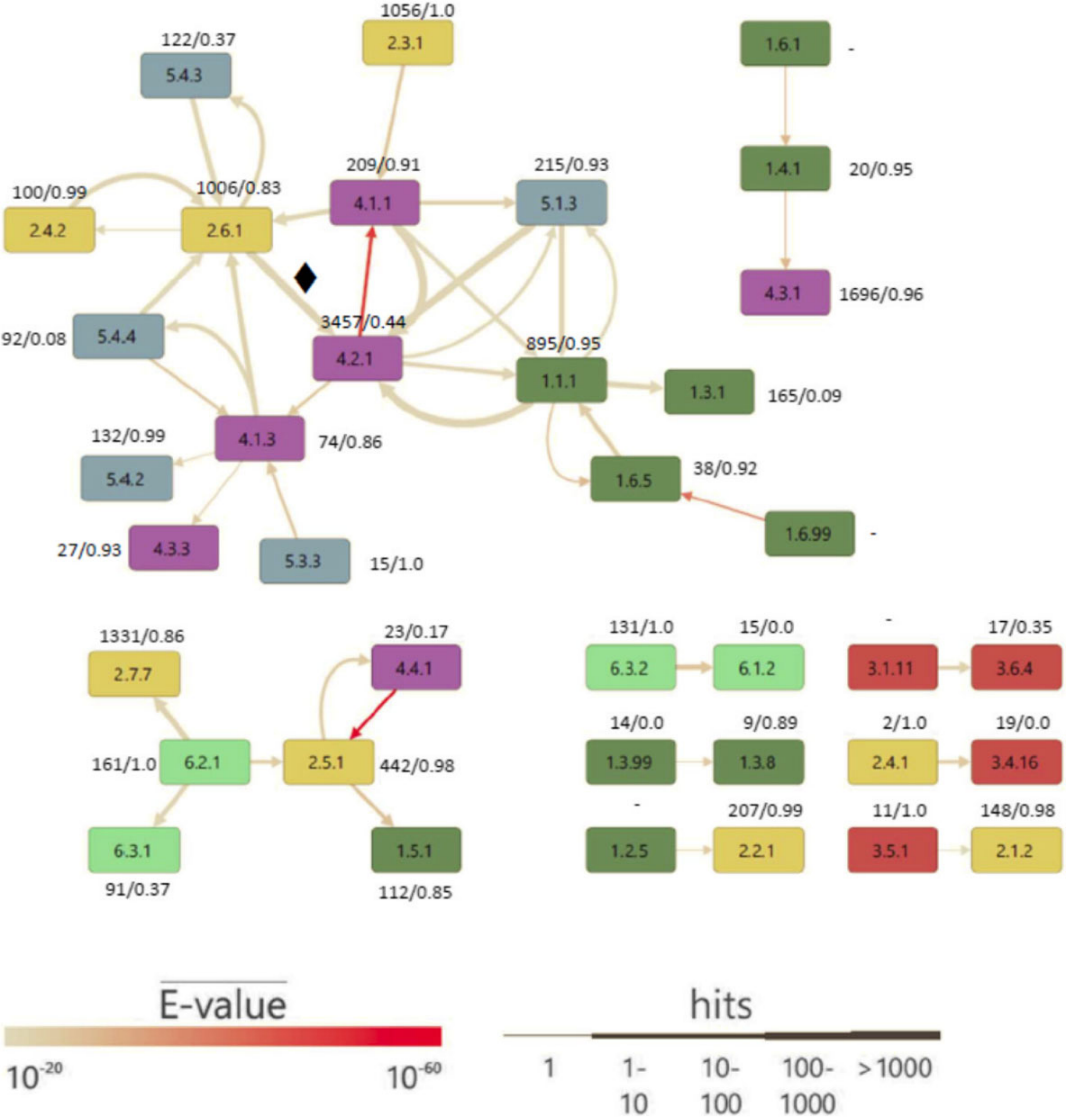
Multifunctionality deduced from homologous PM/SM pairs and determined for EC subdivisions. The nodes represent EC subdivisions and arrows indicate the relation of functional difference PM* → SM*. For example, the arrow 2.6.1 → 4.2.1 signals that PM* homologs of SM* subdivision 4.2.1 belong to EC subdivision 2.6.1; this arrow is marked with a ♦. The width of the arrows represents the number of BLAST hits of enzymes from *enzymes*_SM*_ in *enzymes*_PM*_ and their color the mean E-value; hits were binned as indicated. In addition, for each subdivision, the number of PM* BLAST hits is given and the rate of functional conservation *fc*, which is the fraction of PM* BLAST hits that belong to the same EC subdivision as the SM* queries. Subdivisions that do not occur in *enzymes*_PM*_ are indicated by a “-“

For a more detailed analysis, we computed an analogous network (cutoff 1E-20) on the level of subdivisions (*EC_sd*), which is shown in Fig. 3. For each SM* subdivision EC_sd_SM*, all subdivisions were determined that contained PM* homologs. Thus*,* each directed edge (EC_sd_PM* → EC_sd_SM*) of the network signals an additional reaction chemistry found in some of the PM* homologs. The graph contains six isolated edges and a 2-edge subgraph proposing the limited functional diversity of the corresponding PM homologs. Interestingly, two larger networks arose that subsume enzymes from the EC classes 1, 2, 4, and 5, and from the EC classes 1, 2, 4, and 6, respectively. In the following, we concentrate on the most versatile subdivisions being interconnected in Fig. 3 by reddish and wide arrows. Among oxidoreductases, those that act on the CH-OH group of donors with NAD(+) or NADP(+) as acceptor (EC 1.1.1) and among transferases, transaminases (EC 2.6.1) possess high evolvability. The high functional flexibility of lyases is due to the evolvability of carboxy-lyases (EC 4.1.1), oxo-acid-lyases (EC 4.1.3) and hydro-lyases (EC 4.2.1) and among isomerases, this holds for racemases and epimerases acting on carbohydrates (EC 5.1.3). The lowest functional conservation *fc*(*EC_sd*) of the subdivisions with more than 50 PM* hits possess oxidoreductases acting on CH-CH groups (EC 1.3.1, *fc* = 0.09), hydrolyases (EC 4.2.1, *fc* = 0.44), isomerases transferring amino groups (EC 5.4.3, *fc* = 0.37), isomerases transferring hydroxy groups (EC 5.4.4, *fc* = 0.08), and amide synthases (EC 6.3.1, *fc* = 0.37). In summary, Fig. 3 confirms that the most drastic changes of reaction chemistry associated with the recruitment for SM* occur in isomerases and lyases, which are known as functionally flexible [27].

One can understand many of these multifunctionalities by comparing the substrates and the chemistry of the enzymes. For example, the common substrate of the oxo-acid lyase TrpE (EC 4.1.3.27) and the intermolecular transferase ICS (EC 5.4.4.2) is chorismate and the interconversion of these two enzymatic functions has been demonstrated recently [11]. An other example are the SM* enzyme 2,3-dihydroxybenzoate-AMP ligase (EC 2.7.7.58) that transfers 2,3-dihydroxybenzoate onto ATP and the PM* enzyme o-succinylbenzoyl-coenzyme A synthetase (EC 6.2.1.26) that transfers 2-succinylbenzoate onto ATP. Thus, in both reactions a carboxylic acid substrate is transferred to ATP to give an acid-adenylate.

### A compilation of genomic neighborhoods that support the detailed characterization of homologous PM/SM enzyme pairs

For a direct comparison, those pairs of homologous enzymes are of great interest that are located in the genome of one species and contribute to PM or SM, respectively. In order to make possible a detailed analysis for the user, we restricted the analysis to those 339 BGCs that are annotated in KEGG and named this set *BGC*_*KEGG*_. These BGCs contain 4856 gene products; according to their GO terms [29] 3156 are enzymes; 937 have assigned one of 396 different EC numbers. Based on KEGG annotations and GO terms, we identified all enzymes *enzymes*_*BGC*_*KEGG*_^*species*^ from a single *BGC*_*KEGG*_. Then, the full genome, *i. e.* the DNA sequence of the respective *species* was scanned for homologs of each set *enzymes*_*BGC*_*KEGG*_^*species*^ by using tblastn with a cutoff of 1E-20 and all BLAST hits were added to a BGC-specific html-page.

Each enzyme from *enzymes*_*BGC*_*KEGG*_^*species*^ may possess - in the same genome - several homologs and it is difficult to decide for each BLAST hit *put*_*PM*_ whether it is part of PM or SM, since the functional annotation of a single enzyme may be misleading. As a first additional clue, the label “P” (indicating a possible contribution to PM) was assigned to each gene product, if at least one element of *enzymes*_PM*_ had the same EC number. The label “S” (indicating a possible contribution to SM) was assigned, if KEGG mapped this enzyme function to the species-specific pathway “Biosynthesis of secondary metabolites”. Moreover, the genomic neighborhood of a *put*_*PM*_ may assist the user with classification, because in bacteria, more than 50% of the genes are organized in operons and the gene products are often involved in the same functional pathway [30]. Thus, each *put*_*PM*_-specific ±10 gene neighborhood was added to the html-pages as an additional block of information. These neighbors were further annotated by means of KEGG data and for each gene, a link to the respective KEGG GENES database entry was implemented, which allows for a rapid access to the comprehensive annotation deposited there. To provide further support for the contribution of *put*_*PM*_ to metabolic pathways, the KEGG PATHWAY annotation of the ±10 and the ±2 gene neighborhood of each *put*_*PM*_ were summarized. These two numbers were chosen, because the average operon length deduced for 42 bacterial species is three to four genes and in the genome of the typical bacterium *E. coli*, more than 95% of all operons are shorter than ten genes [31]. Taken together, a genomic neighborhood annotated predominantly with “P” encodes most likely a PM pathway and one can further corroborate this hypothesis by assessing the corresponding KEGG PATHWAY annotations. Combining these data, one can identify such candidates *put*_*PM*_, whose PM membership is highly plausible.

To illustrate the usefulness of these annotations, we detail four cases. Table 3 represents part of the html-page related to BGC000309. This MIBiG cluster specifies the bacillibactin biosynthetic gene cluster (SM) from *Bacillus subtilis*. It contains the gene bsu:BSU_31990, whose product is annotated as an isochorismate synthase (EC 5.4.4.2). One *B. subtilis* homolog with an E-value of 5E-26 is bsu:BSU00740; the gene product is annotated as PabB (EC 2.6.1.85) and is a subunit of the heterodimeric para-aminobenzoate synthase involved in folate biosynthesis (PM). 13 gene products encoded within the corresponding ±10 gene neighborhood of *pab*B have assigned a “P” and not more than two an “S”. Six genes of the ±10 and 3 of the ±2 neighborhood are involved in folate biosynthesis. In summary, these annotations make clear that this DhbC homolog, named PabB, is a PM enzyme.

**Table 3:** Annotation of the ±10 genomic neighborhood of *pab*B from *B. subtilis*

BGC000309; SM* bsu:BSU31990 Isochorismate synthase DhbC (EC 5.4.4.2) ↔ PM bsu:BSU00740 PabB; E-value 5.0E-26			
E_PM	K_SM	KEGG Annotation	Pathways in ±10/±2 Nh
P		bsu:BSU00640 spoIIE; stage II sporulation protein E (EC 3.1.3.16)	6/3 bsu00790 Folate biosynthesis 2/1 bsu01110 Biosynthesis of secondary metabolites 1/1 bsu01130 Biosynthesis of antibiotics 1/1 bsu00270 Cysteine and methionine metabolism 1/1 bsu00920 Sulfur metabolism 1/0 bsu00970 Aminoacyl-tRNA biosynthesis 1/0 bsu01200 Carbon metabolism 1/0 bsu00770 Pantothenate and CoA biosynthesis 1/0 bsu00230 Purine metabolism 1/1 bsu01230 Biosynthesis of amino acids
		bsu:BSU00650 yabS; hypothetical protein; K07114 Ca-activated chloride channel homolog	
P		bsu:BSU00660 yabT; serine/threonine protein kinase (EC 2.7.11.1)	
P		bsu:BSU00670 tilS; tRNA(ile)-lysidine synthase; K04075 tRNA(Ile)-lysidine synthase[EC 6.3.4.19]	
P	S	bsu:BSU00680 hprT; hypoxanthine-guanine phosphoribosyltransferase (EC 2.4.2.8)	
		bsu:BSU00690 ftsH; ATP-dependent zinc metalloprotease FtsH (EC 3.4.24	
P		bsu:BSU00700 coaX; type III pantothenate kinase (EC 2.7.1.33)	
		bsu:BSU00710 hslO; 33 kDa chaperonin; K04083 molecular chaperone Hsp33	
		bsu:BSU00720 yacD; peptidyl-prolyl cis-trans isomerase	
P	S	bsu:BSU00730 cysK; cysteine synthase (EC 2.5.1.47)	
P		bsu:BSU00740 pabB; para-aminobenzoate synthase component I (EC 2.6.1.85)	
P		bsu:BSU00750 pabA; para-aminobenzoate/anthranilate synthase component II (EC 2.6.1.85)	
P		bsu:BSU00760 pabC; aminodeoxychorismate lyase (EC 4.1.3.38	
P		bsu:BSU00770 sul; dihydropteroate synthase (EC 2.5.1.15	
P		bsu:BSU00780 folB; dihydroneopterin aldolase (EC 4.1.2.25	
P		bsu:BSU00790 folK; 2-amino-4-hydroxy-6-hydroxymethyldihydropteridine pyrophosphokinase	
		bsu:BSU00800 yazB; XRE family transcriptional regulator	
		bsu:BSU00810 dusB; tRNA-dihydrouridine synthase (EC 1.-.-.-)	
P		bsu:BSU00820 lysS; lysine--tRNA ligase	
		bsu:BSU00830 ctsR; transcriptional regulator CtsR	
		bsu:BSU00840 mcsA; hypothetical protein; K19411 protein arginine kinase activator	

The first line gives the name of the MIBiG cluster containing the considered SM* enzyme, the annotation of the SM* and the related putative PM enzyme from the same genome, and the BLAST E-value resulting from the comparison of the corresponding two protein sequences

The following lines characterize the ±10 genomic neighbourhood of the putative PM enzyme. A “P” in column “E_PM” indicates that this enzyme function, *i. e*. EC number, occurs in *enzymes*_PM*_ and an “S” in column “K_SM” indicates that KEGG assigned this enzyme function to the pathway “Biosynthesis of secondary metabolites”. The column named “KEGG Annotation” lists KEGG-ID, function and EC number of the gene products. The column named “Pathways in ±10/±2 Nh” lists the number of genes from the corresponding two neighborhoods of the putative PM enzyme that belong to the listed KEGG pathways. For this table, the gene annotations taken from the respective html-page were shortened for the sake of brevity

As explained in the Introduction, the ICS DhbC possesses a further PM homolog, which is an anthranilate synthase (EC 4.1.3.27) [11]. As expected, our analysis identified this SM/PM enzyme pair as well: A second DhbC homolog with an E-value of 3E-20 is bsu:BSU22680 and its neighborhood is depicted in Table 4. This gene product is one subunit of the heterodimeric anthranilate synthase involved in tryptophan biosynthesis. However, the annotation given in Table 4 illustrates the difficulties of assigning the function of individual gene products to PM or SM, because 17 entries of the *trp*E neighborhood are labeled with a “P” and 16 with an “S”. Following the link of bsu:BSU22680 (*trp*E) and clicking the “Genome map” button on the html-page for this KEGG GENES entry, one can easily verify that this neighborhood that contains the genes *trp*A – *trp*E is the *trp* operon of *B. subtilis*. Thus, the KEGG PATHWAY annotation “Biosynthesis of secondary metabolites” assigned to 16 gene products of this neighborhood is misleading whereas the less frequently assigned annotation “Phenylalanine, tyrosine and tryptophan biosynthesis“ is correct.

**Table 4:** Annotation of the ±10 genomic neighborhood of *trp*E from *B. subtilis*

BGC0000309; SM* bsu:BSU31990 Isochorismate synthase DhbC (EC 5.4.4.2) ↔ PM bsu:BSU22680 trpE; E-value 3.0E-20			
E_PM	K_SM	KEGG Annotation	Pathways in ±10/±2 Nh
		bsu:BSU22590 ypiA; TPR repeat-containing protein YpiA	16/5 bsu01110 Biosynthesis of secondary metabolites 13/5 bsu01130 Biosynthesis of antibiotics 12/5 bsu00400 Phenylalanine, tyrosine and tryptophan biosynthesis 12/5 bsu01230 Biosynthesis of amino acids 2/0 bsu00401 Novobiocin biosynthesis 2/0 bsu00260 Glycine, serine and threonine metabolism 2/0 bsu00900 Terpenoid backbone biosynthesis 1/0 bsu02020 Two-component system 1/0 bsu00790 Folate biosynthesis 1/0 bsu02030 Bacterial chemotaxis 1/0 bsu00240 Pyrimidine metabolism 1/0 bsu00230 Purine metabolism 1/0 bsu00340 Histidine metabolism 1/0 bsu00360 Phenylalanine metabolism 1/0 bsu00130 Ubiquinone and other terpenoid-quinone biosynthesis 1/0 bsu00350 Tyrosine metabolism
P	S	bsu:BSU22600 aroE; 3-phosphoshikimate 1-carboxyvinyltransferase (EC 2.5.1.19)	
P	S	bsu:BSU22610 tyrA; prephenate dehydrogenase (EC 1.3.1.12)	
P	S	bsu:BSU22620 hisC; histidinol-phosphate aminotransferase (EC 2.6.1.9)	
P	S	bsu:BSU22630 trpA; tryptophan synthase alpha chain (EC 4.2.1.20)	
P	S	bsu:BSU22640 trpB; tryptophan synthase beta chain (EC 4.2.1.20)	
P	S	bsu:BSU22650 trpF; N-(5'-phosphoribosyl)anthranilate isomerase (EC 5.3.1.24)	
P	S	bsu:BSU22660 trpC; indole-3-glycerol phosphate synthase (EC 4.1.1.48)	
P	S	bsu:BSU22670 trpD; anthranilate phosphoribosyltransferase (EC 2.4.2.18)	
P	S	bsu:BSU22680 trpE; anthranilate synthase component 1 (EC 4.1.3.27)	
P	S	bsu:BSU22690 aroH; chorismate mutase AroH (EC 5.4.99.5)	
P	S	bsu:BSU22700 aroB; 3-dehydroquinate synthase (EC 4.2.3.4)	
P	S	bsu:BSU22710 aroF; chorismate synthase (EC 4.2.3.5)	
P		bsu:BSU22720 cheR; chemotaxis protein methyltransferase (EC 2.1.1.80)	
P	S	bsu:BSU22730 ndk; nucleoside diphosphate kinase (EC 2.7.4.6)	
P	S	bsu:BSU22740 hepT; heptaprenyl diphosphate synthase component 2 (EC 2.5.1.30)	
	S	bsu:BSU22750 ubiE; demethylmenaquinone methyltransferase (EC 2.1.1.-)	
P	S	bsu:BSU22760 hepS; heptaprenyl diphosphate synthase component 1 (EC 2.5.1.30)	
		bsu:BSU22770 mtrB; transcription attenuation protein MtrB	
P		bsu:BSU22780 folE; GTP cyclohydrolase 1 (EC 3.5.4.16)	
		bsu:BSU22590 ypiA; TPR repeat-containing protein YpiA	

The first line gives the name of the MIBiG cluster containing the considered SM* enzyme, the annotation of the SM* and the related putative PM enzyme from the same genome, and the BLAST E-value resulting from the comparison of the corresponding two protein sequences

The following lines characterize the ±10 genomic neighbourhood of the putative PM enzyme. A “P” in column “E_PM” indicates that this enzyme function, *i. e*. EC number, occurs in *enzymes*_PM*_ and an “S” in column “K_SM” indicates that KEGG assigned this enzyme function to the pathway “Biosynthesis of secondary metabolites”. The column named “KEGG Annotation” lists KEGG-ID, function and EC number of the gene products. The column named Pathways in ±10/±2 Nh” lists the number of genes from the corresponding two neighborhoods of the putative PM enzyme that belong to the listed KEGG pathways. For this table, the gene annotations taken from the respective html-page were shortened for the sake of brevity

The recruitment of *trp* genes for SM is further documented by the results for BGC0000315, which is the calcium-dependent antibiotic biosynthetic gene cluster from *Streptomyces coelicolor* (strain ATCC BAA-471/A3(2)/M145). The respective html-page shows that this BGC contains the genes *trp*C2, *trp*D2, and *trp*E, and additional copies of *trp* genes can be found in the rest of this genome. Our annotation of the SCO7691 neighborhood shown in Table 5 makes clear that this gene does not encode a PM enzyme. Thus, due to the fact that each entry from *enzymes*_*BGC*_*KEGG*_^*species*^ can possess several homologs in the same genome, the neighborhoods which we supply have to be analyzed carefully by the user in order to assign enzymes to PM or SM.

**Table 5:** Annotation of the ± 10 genomic neighborhood of gene SCO7691 from *S. coelicolor*

BGC0000315; SM* sco:SCO3214 Anthranilate synthase component 1 (EC 4.1.3.27) ↔ SC4C2.26; lyase; K04781 salicylate synthetase; E-value 7.0E-36			
E_PM	K_SM	KEGG Annotation	Pathways in ±10/±2 Nh
		sco:SCO7681; AMP-binding ligase	2/1 sco01053 Biosynthesis of siderophore group 1/1 sco01130 Biosynthesis of antibiotics 1/1 sco01110 Biosynthesis of secondary metabolites 1/0 sco00562 Inositol phosphate metabolism
		sco:SCO7682; non-ribosomal peptide synthase	
		sco:SCO7683; non-ribosomal peptide synthase	
		sco:SCO7684; hypothetical protein	
		sco:SCO7685; hypothetical protein	
		sco:SCO7686; cytochrome P450	
		sco:SCO7687; thioesterase	
		sco:SCO7688; hypothetical protein	
		sco:SCO7689; ABC transporter ATP-binding protein	
		sco:SCO7690; ABC transporter ATP-binding protein	
	S	sco:SCO7691; lyase; K04781 salicylate synthetase	
		sco:SCO7692; hypothetical protein	
		sco:SCO7693; oxidoreductase	
		sco:SCO7694; TetR family transcriptional regulator	
		sco:SCO7695; hypothetical protein	
		sco:SCO7696; MarR family transcriptional regulator	
		sco:SCO7697; hydrolase; K01083 3-phytase (EC 3.1.3.8)	
		sco:SCO7698; MerR family transcriptional regulator	
		sco:SCO7699; nucleotide-binding protein	
		sco:SCO7700; cyclase; (EC:4.2.3.118)	
		sco:SCO7701; methyltransferase; (EC:2.1.1.255)	

The first line gives the name of the MIBiG cluster containing the considered SM* enzyme, the annotation of the SM* and the related putative PM enzyme from the same genome, and the BLAST E-value resulting from the comparison of the corresponding two protein sequences

The following lines characterize the ±10 genomic neighbourhood of the putative PM enzyme. A “P” in column “E_PM” indicates that this enzyme function, *i. e*. EC number, occurs in *enzymes*_PM*_ and an “S” in column “K_SM” indicates that KEGG assigned this enzyme function to the pathway “Biosynthesis of secondary metabolites”. The column named “KEGG Annotation” lists KEGG-ID, function and EC number of the gene products. The column named Pathways in ±10/±2 Nh” lists the number of genes from the corresponding two neighborhoods of the putative PM enzyme that belong to the listed KEGG pathways. For this table, the gene annotations taken from the respective html-page were shortened for the sake of brevity

**Fig. 4:**
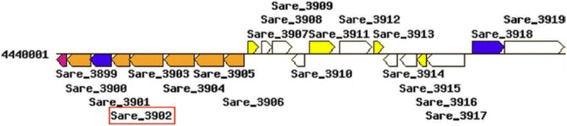
KEGG genome map for the neighborhood of gene Sare_3902 from *S. arenicola*. The picture was created by using the “Genome map” function of the KEGG gene entry Sare_3902. KEGG uses the following color code to fill the arrows representing genes: amino acid metabolism (orange), metabolism of cofactors and vitamins (pink), energy metabolism (violet), environmental information processing (yellow), unclassified (white). Sare_3902 codes for a 4-oxalocrotonate decarboxylase; compare Table 6

A further example for a *bona fide* pair of homologous PM/SM enzymes is shown in Table 6. BGC0000333 is the cyclomarin biosynthetic gene cluster from *Salinispora arenicola* (strain CNS-205). It contains the gene saq:Sare_4569 that codes for a 4-oxalocrotonate decarboxylase (EC 4.1.1.77). The respective genome contains the two homologs saq:Sare_3899 and saq:Sare_3902 that are involved in benzonate and tryptophan degradation. The extended annotation shown in Table 6 strongly suggests that their genomic neighborhood encodes PM enzymes. Figure 4, which was created by clicking the “Genome map” button of saq:Sare_3902, provides further evidence: The graph confirms that the latter two genes are part of an operon containing enzymes from PM, which illustrates the benefits of using KEGG data.

**Table 6:** Annotation of the ±10 genomic neighborhood of Sare_3902 from *S. arenicola* (strain CNS-205)

BGC0000333; SM* saq:Sare_4569 4-oxalocrotonate decarboxylase (EC 4.1.1.77) ↔ PM saq:Sare_3902; E-value 4.0E-47			
E_PM	K_SM	KEGG Annotation	Pathways in ±10/±2 Nh
		saq:Sare_3892 aminopeptidase N (EC 3.4.11.2)	8/2 saq00380 Tryptophan metabolism 6/4 saq01120 Microbial metabolism in diverse environments 5/4 saq00622 Xylene degradation 5/4 saq00362 Benzoate degradation 5/4 saq01220 Degradation of aromatic compounds 4/3 saq00360 Phenylalanine metabolism 4/3 saq00621 Dioxin degradation 1/0 saq00643 Styrene degradation 1/0 saq00330 Arginine and proline metabolism 1/1 saq00620 Pyruvate metabolism 1/1 saq00650 Butanoate metabolism 1/0 saq00480 Glutathione metabolism 1/0 saq00627 Aminobenzoate degradation
		saq:Sare_3893 conserved hypothetical protein	
		saq:Sare_3894 conserved hypothetical protein	
P		saq:Sare_3895 Amidase; K01426 amidase (EC 3.5.1.4)	
		saq:Sare_3896 amidohydrolase 2 (EC 4.1.1.45)	
		saq:Sare_3897 3-hydroxyanthranilate 3,4-dioxygenase (EC 1.13.11.6)	
		saq:Sare_3898 Endoribonuclease L-PSP (EC 3.5.99.5)	
P		saq:Sare_3899 4-oxalocrotonate decarboxylase (EC 4.1.1.77)	
P		saq:Sare_3900 pyruvate carboxyltransferase	
P		saq:Sare_3901 Acetaldehyde dehydrogenase	
P		saq:Sare_3902 4-oxalocrotonate decarboxylase (EC 4.1.1.77)	
		saq:Sare_3903 aldehyde dehydrogenase	
		saq:Sare_3904 Kynurenine 3-monooxygenase (EC 1.14.13.9)	
P		saq:Sare_3905 kynureninase; K01556 kynureninase (EC 3.7.1.3)	
		saq:Sare_3906 tryptophan 23-dioxygenase	
		saq:Sare_3907 transcriptional regulator	
		saq:Sare_3908 conserved hypothetical protein	
		saq:Sare_3909 peptidase C60 sortase A and B	
		saq:Sare_3910 HNH endonuclease	
		saq:Sare_3911 MscS Mechanosensitive ion channel	
		saq:Sare_3912 major facilitator superfamily MFS_1	

The first line gives the name of the MIBiG cluster containing the considered SM* enzyme, the annotation of the SM* and the related putative PM enzyme from the same genome, and the BLAST E-value resulting from the comparison of the corresponding two protein sequences

The following lines characterize the ±10 genomic neighbourhood of the putative PM enzyme. A “P” in column “E_PM” indicates that this enzyme function, *i. e*. EC number, occurs in *enzymes*_PM*_ and an “S” in column “K_SM” indicates that KEGG assigned this enzyme function to the pathway “Biosynthesis of secondary metabolites”. The column named “KEGG Annotation” lists KEGG-ID, function and EC number of the gene products. The column named “Pathways in ±10/±2 Nh” lists the number of genes from the corresponding two neighborhoods of the putative PM enzyme that belong to the listed KEGG pathways. For this table, the gene annotations taken from the respective html-page were shortened for the sake of brevity

A compilation of all *BGC*_*KEGG*_ html-pages can be downloaded from https://www.bioinf.ur.de. To create this version, we considered all BLAST hits with an E-value ≤ 1E-20. If one is interested to search homologs of *BGC*_*KEGG*_ gene products more sensitively, one only has to follow the links we have integrated. They lead to the respective KEGG GENES entries and one can initiate a BLAST search with a user-defined set of genomes (or a single one) by means of the “DB search” function of KEGG.

## Discussion

### The broad functional transition zone that links PM and SM impedes the analysis of SM evolution

Assigning enzyme functions to PM or SM is hampered by several facts. As already mentioned, biosynthetic compounds like lipids or polysaccharides are “essential” for every organism, but the synthesis of some of them in SM makes possible a specific interaction of the producing organisms with their environment [9]. Thus, for these enzymatic functions, the assignment to PM or SM is a species-specific problem. Moreover, enzymes like those of the rhamnose biosynthesis pathway supply precursors for PM and SM [32] and the products of tryptophan biosynthesis and other PM pathways are utilized in SM. For these cases, it is difficult to draw the line between PM and SM.

Due to these circumstances, we decided to analyze two subsets, for which PM or SM assignment is highly reliable. However, the surrogates (*enzymes*_PM*_ and *enzymes*_SM*_) which we compiled, have their specific drawbacks: Most likely, the number of enzymatic functions contributing to bacterial PM and SM is larger than estimated here. As a consequence, the number of SM enzymes recruited from PM is most likely underestimated and the stringent BLAST cutoff [33], which we used to minimize false positives, might additionally contribute to this effect. Thus, we have estimated a lower limit for the functional flexibility of protein folds.

However, despite these limitations, we could deduce several important characteristics of SM enzymes: *i*) From the bird’s eye view the spectra of enzymatic functions utilized in SM* and PM* are highly similar. *ii*) The finding that 331 SM* functions possess homologous PM* enzymes strongly support the recruitment theory. *iii*) Even if we underestimated the functional flexibility of enzymes, we could underpin the broad spectrum of metabolic neofunctionalization, which is exploited by evolution, both in PM and SM.

### PM/SM pairs represent a large playground to study enzyme evolution, promiscuity, and their regulatory fine-tuning

Usually, PM pathways produce single products. For example, the tryptophan biosynthetic pathway makes only tryptophan. In contrast, pathways of SM are diversity-oriented and may synthesize up to 100 products [34] which seems puzzling at first glance. However, a decent biological activity is a rare property of a product [9] and thus evolution favors organisms able to generate in SM chemical diversity at low cost. It follows that organisms producing many different compounds improve their fitness, because the number of synthesized products increases the probability that some are biologically active. Along these lines, the wide-spread use of branched and matrix biosynthetic pathways that makes it difficult to distinguish PM and SM enzymes, helps to share metabolic and genetic costs [34].

A further route leading to a widened chemical diversity is the promiscuity of SM enzymes. It has been made plausible that SM enzymes emerged through early gene duplication followed by mutations that broadened substrate selection and flattened activation barriers [35] at the expense of efficiency [20]. Interestingly, it has been shown that promiscuity can be achieved without compromising efficiency [36] and directed evolution and combinatorial engineering approaches are winning strategies to optimize the production of secondary metabolites [37, 38]. Due to their broader substrate specificity, we propose to consider SM enzymes also for more general enzyme design projects beyond secondary metabolism. Such a strategy has great potential because for at least 391 enzymatic functions that are also relevant in PM, we found at least one enzyme in secondary pathways of bacterial species.

These SM generalists are often slow, because such a catalytic inefficiency is beneficial, *e. g*., to avoid competition with primary metabolism [20]. A fine-tuning of enzymatic activities competing for substrates is most critical for homologous PM/SM enzymes that are active in the same cell. In order to identify such cases, we analyzed BGC clusters within their genomic contexts. The resulting species-specific compilation of these PM/SM pairs is now an ideal basis for a further *in silico* analysis and the design of biochemical experiments needed for the detailed characterization of these enzymes and their regulation.

## Conclusions

Secondary, *i. e*., specialized metabolites produced by bacteria exhibit enormous structural variation and possess a vast range of biological activities. Interestingly, the reaction chemistry used in BGCs to produce these metabolites does not differ drastically from PM. Only few EC subdivisions (*i. e*. reaction chemistries) are overrepresented in BGCs and for at least 331 enzyme functions found in bacterial BGCs, homologs exist in PM. The functional spectra of homologs are similar, indicating that the evolvability of protein folds is key for establishing novel enzymatic functions, both in PM and SM. Most interesting cases of functional interconversion can be found by scanning the html-pages we provide for each BGC. These homologous PM/SM enzyme pairs are active in the same species and their co-existence may require specific regulatory elements or a fine-tuning of function.

## Methods

### Software and databases

Programs were written in Java (https://java.com/download). Java-based APIs (JAPIs) were used to access the databases BRENDA (SOAP API at http://www.brenda-enzymes.org/soap.php), KEGG (REST-API at http://www.kegg.jp/kegg/rest/keggapi.html), and UniProt (API at http://www.ebi.ac.uk/uniprot/remotingAPI). The full genomes of the species listed in Table 1 were assessed by means of KEGG to compile *enzymes*_PM*_. The MIBiG dataset (version 1.0, http://mibig.secondarymetabolites.org/repository.html) was downloaded and analyzed locally to deduce SM* enzymes.

For an unequivocal assignment of function, the above databases were scanned to deduce for each enzyme EC numbers by means of the UniProt ID. Only those enzymes were added to *enzymes*_PM*_ or *enzymes*_SM*_, respectively, that had assigned an EC number. To avoid ambiguities, enzymes that were annotated with more than one EC number were eliminated as well; among them were 77 SM* enzymes.

To search for homologs, tblastn and blastp of BLAST [39] were used; for BLASTing KEGG databases, KEGG-BLAST was utilized via the html-page http://www.genome.jp/tools/blast/. Generally, two enzymes were considered as homologous (*i. e.* share the same fold), if the BLAST E-value was ≤ 1E-20. As a control, the cutoff 1E-10 was applied.

**Fig. 5:**
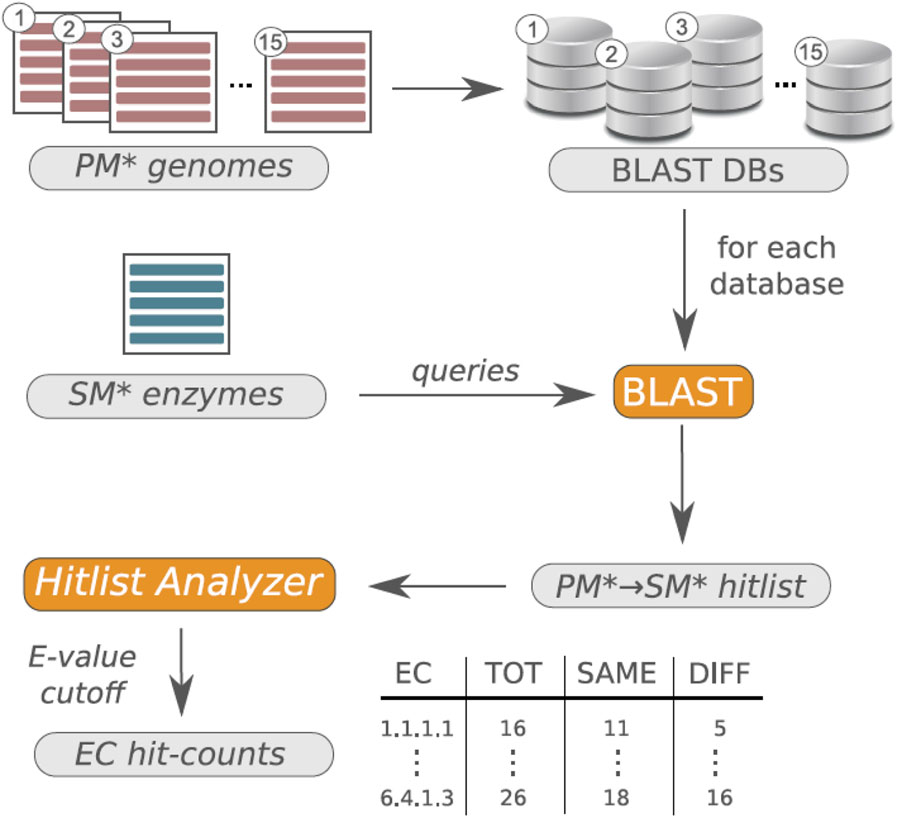
Software architecture for the determination of PM*/SM* homologs. This schema illustrates how the number and the enzyme function of PM* homologs was determined for SM* enzymes. For each of the 15 species listed in Table 1, the genome was downloaded from KEGG and the functional assignment was supplemented by using InterPro and other databases. Subsequently, a BLAST database (DB) was built for each of the genomes. The sequences of the SM* enzymes deduced from the chosen BGCs were then BLASTed against all 15 databases. All BLAST hits were stored in PM* → SM* hitlist that contained all PM* → SM* pairs. Based on the chosen E-value cutoff, our program *Hitlist Analyzer* selected those hits that both had assigned an EC number and compiled the output table *EC hits-counts*. This table contained for each EC number the number of PM* hits (TOT) and the number of PM* hits having assigned the same (SAME) and a different (DIFF) EC number. These raw data were further processed to determine frequencies and related parameters for EC numbers, subdivisions, and classes

To assess the functional variety of enzyme families, BLAST was used to identify for each *query*_*k*_ homologs of all query sequences *queries* = {*query*_*k*_} in a set of reference sequences *references* = {*ref*_*l*_}. The XML output of BLAST was parsed and analyzed using Python 2.7 [40] and Biopython [41]. Only those hits *ref*_*l*_ that deviated in length by not more than 30% from the sequence *query*_*k*_ were further processed. *query*_*k*_ entries were sorted according to their EC number and the corresponding EC number distribution of their hits was determined and normalized to create the sets *EC_cl* and *EC_sd*. For a comparison of SM* and PM* enzymes, the *queries* were all *enzymes*_SM*_ sequences and the references were the sequences from *enzymes*_PM*_. Figure 5 illustrates the software protocol for this case. To determine functional variety in PM*, for each of the 15 query sets $enzyme{s}_{genom{ e}_i}$, the reference set was $reference{s}_i=\left\{ e nzyme{s}_{PM*}\backslash enzyme{s}_{genom{ e}_i}\right\}$. To determine functional variety in SM*, the enzymes of one *BGC*_*i*_ constituted the query sets $enzyme{s}_{BG{ C}_i}$ and the reference sets were $reference{s}_i=\left\{ enzyme{s}_{SM*}\backslash enzyme{s}_{BG{ C}_i}\right\}$. Additional file 4 contains the sequences of *enzymes*_PM*_ and Additional file 5 the sequences of *enzymes*_SM*_.

Relationships between EC classes (EC subdivisions) were visualized as directed graphs using Cytoscape 3.3 and the yFiles circular layout [28]. Edge widths correspond to the number of the respective BLAST hits; edge colors correspond to the mean E-value of these pairs.

## Additional files

Additional file 1: Table S1. Table in Excel format listing the occurrence of EC numbers, EC subclasses, and of EC subdivisions in *enzymes*_*PM**_ and *enzymes*_*SM**_. (XLSX 114 kb)
Additional file 2: Table S2. Table in Excel format listing the number of homologous BLAST hits found in *enzymes*_*PM**_ for all enzymes represented in *enzymes*_*SM**_. Hits are added according to EC numbers and EC subdivisions. (XLSX 29 kb)
Additional file 3: Figure S1. Figure in PDF format showing the analysis of neofunctionalization based on the BLAST cutoff 1E-10. (PDF 146 kb)
Additional file 4: 15 multiple Fasta files (1 per genome, compare Table 1) containing the sequences of the data set *enzymes*_*PM**_. The genomes are named according to KEGG nomenclature. (ZIP 7193 kb)
Additional file 5: 1005 multiple Fasta files (one per BGC that comprises enzymes) containing the sequences of the data set *enzymes*_*SM**_. The BGC are named according to MIBiG nomenclature. (ZIP 6333 kb)
