# Supplementary material for: Assessing in silico the recruitment and functional spectrum of bacterial enzymes from secondary metabolism
Source: BMC Evol Biol. 2017 Jan 26;17:36. doi: 10.1186/s12862-017-0886-2 (PMC5270213; doi:10.1186/s12862-017-0886-2)

**Supplementary Figure 1 – Multifunctionality deduced from homologous PM*/SM* pairs and determined for EC classes; BLAST cutoff 1E-10**

The nodes represent the six EC classes and arrows indicate the relation of functional difference PM* → SM*. The width of the arrows represents the number of BLAST hits of enzymes from $enzymes_{SM*}$ in $enzymes_{PM*}$ and their color the mean E-value; hits were binned as indicated. In addition, for each class, the number of PM BLAST hits is given and the rate of functional conservation *fc*, which is the fraction of PM BLAST hits that belong to the same EC class as the SM queries. The class EC 1 subsumes oxidoreductases that catalyze oxidation/reduction reactions and EC 2 transferases that transfer functional groups. EC 3 consists of hydrolases that catalyze the formation of two products from a substrate by hydrolysis and EC 4 contains lyases that catalyze the non-hydrolytic addition or removal of groups. The isomerases of EC 5 catalyze the intramolecular rearrangement within a single molecule and the ligases of EC 6 join together two molecules under consumption of ATP or similar triphosphates. The arrow 6 → 5 is marked with a ♦ signals and is the only additional case of neofuctionalzation that was not detected by using the cutoff 1E-20 shown in Fig. 2.

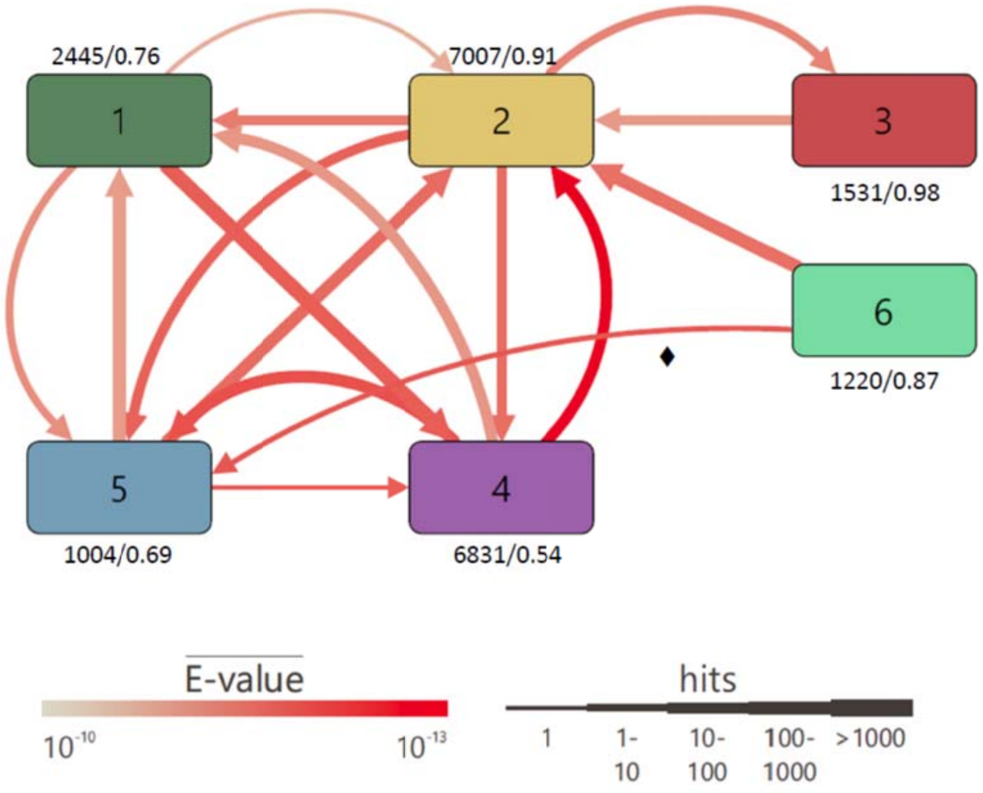

Supplement: Additional file 3: Figure S1. — Figure in PDF format showing the analysis of neofunctionalization based on the BLAST cutoff 1E-10. (PDF 146 kb) [file 12862_2017_886_MOESM3_ESM.pdf]
